# Feasibility evaluation of a pain self-management app-based intervention among older people living with arthritic pain: study protocol

**DOI:** 10.1186/s40814-019-0442-5

**Published:** 2019-04-25

**Authors:** Priyanka Bhattarai, Toby R. O. Newton-John, Jane L. Phillips

**Affiliations:** 1 University of Notre Dame Australia, School of Nursing, Sydney, NSW Australia; 2 University of Technology Sydney, Sydney, NSW Australia; 3 University of Notre Dame Australia, Sydney, NSW Australia

**Keywords:** Older adults, Pain management, Arthritis, Smartphone, App, Technology

## Abstract

**Background:**

Optimal management of chronic arthritic pain experienced by older adults involves applying active self-management strategies every day. Cost-effective and innovative strategies to help build older people’s pain self-management capability are required. This study protocol is designed to evaluate the feasibility, acceptability, and preliminary outcomes of a pain self-management app among older people living in the community with arthritic pain.

**Methods/design:**

This is a phase I feasibility study. A pre-post test study design will be used to trial a freely available pain self-management app named Rheumatoid Arthritis Information Support and Education (“RAISE”) for 14 days. Thirty community-dwelling older people living with arthritic pain who use a smartphone will be recruited from (1) various community-based social clubs/organizations/groups or (2) via Facebook groups with potentially high number of older members. In addition, snowballing sampling approach will also be utilized.

These participants will trial the RAISE app, which was selected following a systematic evaluation of all available chronic pain apps by the investigator team. A face-to-face or telephone-based meeting will be organized with all consenting participants in order to seek their informed consent, download and set up the intervention app on their mobile device, be provided with app training, and complete the pre-test data (Time 1 (T1)). Participants will be asked to use the RAISE app as desired for 14 days. Post-test data collection (Time 2 (T2)) will occur on day 15. Data collected includes participant’s demographic and clinical information, pain scores, pain self-efficacy, and online technology self-efficacy. Participants will be invited to take part in a semi-structured telephone interview at T2 to explore their experiences of using the app.

An evaluation of patterns of app use, recruitment, retention, attrition rates, and analysis of the missing data will inform the study and intervention feasibility. Preliminary outcomes are participant’s pain intensity and interference, pain self-efficacy, and online technology self-efficacy.

**Discussion:**

This study will help us better understand the feasibility and acceptability of using this novel intervention among community-dwelling older people living with arthritic pain. The results will also help inform future pain app studies.

**Trial registration:**

Australia New Zealand Clinical Trials Registry: ACTRN12617000921381.

## Background

Unrelieved pain is one of the most common health conditions affecting the majority of older people (those aged over 65 years) [1–3]. Globally, about 70% of older adults suffer from arthritis-related pain [2, 3]. Although the global economic impact of arthritis remains unknown, it is estimated to cost high-income countries between 1–2.5% of their gross national product [4]. As most older people live in the community, self-management strategies are central to improving their unrelieved chronic pain and minimizing the adverse impact of pain on their lives [5, 6]. Active self-management involves (i) medical management (i.e., medication adherence, dietary modification), (ii) behavior modification (i.e., modifying activities of living, physical, and recreational activities), and (iii) managing emotion (i.e., dealing with fear, frustration, and anger) [7]. Building patients’ capacity to self-manage pain is underpinned by effective instruction, education, and support, augmented with regular medical, nursing, and/or allied health follow-up. Self-management strategies are central to maximizing effective coping with pain and minimizing the adverse impact of pain on older people’s lives [5, 6]. While structured pain self-management programs have traditionally been grounded in face-to-face coaching approaches, there has been a growing interest in the use of technology-mediated self-management intervention to promote and support self-management.

Although younger people outnumber older people in terms of technology engagement, the uptake of new technology among older people is increasing rapidly [8]. The proportion of older people using smartphones has more than doubled since 2014 [9], with over 40% of people aged over 65 years now owning a smartphone [10]. A growing proportion of older people regularly use the internet and source online health information [8]. While digital technology uptake among older people of today is increasing and is yet to reach saturation, the next generations of older people (“baby boomers”) who have grown old with digital technology are likely to be even familiar users. Given this reality, there will be more opportunities to use digital technology-facilitated approaches to reach and meet the needs of the world’s rapidly growing and aging population.

Since the introduction of the smartphone in 2007, its subsequent widespread adoption has fueled the development of a range of health-related applications (apps) that can be accessed from these computerized mobile handsets [11]. Smartphone apps use software that interact with users on an individual basis [12]. Health-related apps form a significant proportion of all apps. Although exercise and wellness apps form the major proportion of the health app landscape, self-management apps for chronic conditions, including pain, are growing [13]. There are currently over 350 pain self-management apps offering pain assessment recording, pain information, and pain self-management plans available on the internet, and this number is expected to increase [14, 15]. While several recent systematic reviews have evaluated the quality of currently available pain-related apps [14–17], only one review has examined the quality and usability of arthritic pain self-management apps for older people [13]. This review identified that only 4 of the available 373 pain apps offered pain self-management advice and support in accordance with the Stanford Arthritis Self-Management Program and reflected current arthritic pain management evidence [13].

There is a paucity of pain app evidence that is applicable to older people, with most studies focusing primarily on younger people’s management of their chronic pain [18–20]. The two app-based interventions tested among older people have both focused on areas outside of pain, such as strength training [21] or falls [22]. As older people with chronic pain indicate a willingness to learn and use smartphones and apps for pain self-management [23, 24], the feasibility, acceptability, and effectiveness of using a pain app to help older people better self-manage their arthritic pain warrant further investigation.

## Aim

The aim of this study is to evaluate the feasibility, acceptability, and preliminary outcomes of a pain self-management app among older people living in the community with arthritic pain.

## Method

### Design

This is a phase I feasibility study. Based on the Medical Research Council’s continuum of evidence structure, this study is classed as a phase I study [25, 26]. Studies classed as phase I are designed to improve the understanding of components of an intervention and their interrelationships. Qualitative testing is recommended for use in these studies so as to facilitate the understanding of how the intervention might work [25]. It is, however, important to note that development and evaluation of a complex intervention is not always a sequential or linear process, and these interventions may work best if they are tailored to local settings instead of a uniform/standardized approach [26].

Feasibility studies are used to determine whether an intervention is appropriate for further evaluation [27]. Adhering to the conceptual framework outlined by Eldridge and colleagues [28], this study could be classed under “feasibility studies that are not pilot studies.” Feasibility studies differ from pilot studies as they are carried out before a main study so as to estimate important parameters required to design the main study, whereas a pilot study is a smaller version of the main study carried out to evaluate if all of the components of the main study can work together [29].

### Participants

This study aims to recruit 30 older people living with arthritic pain, who use a smartphone or a tablet device, and who meet the following inclusion criteria:

- Living in the community;
- Aged 65 years or over;
- Presence of arthritic pain for ≥ 3 months (participant reported);
- Ability to read and write in English;
- Ownership of a smartphone/tablet-computer;
- Ability to give written informed consent

Exclusion criteria include the following:

- Presence of cancer pain
- Under end-of-life care pathways
- Living in an institutional home

### Setting and procedure for recruitment

The screening, recruitment, and consenting process for this study will be carried out by one of the investigators (PB). Prospective participants will be sought from various older people’s club/associations/groups, including Facebook groups across Metropolitan and Regional New South Wales (NSW), Australia. This approach of recruitment is considered because many older Australian utilize their free time keeping active and participating in leisure activities by engaging in social clubs [30]. With over 60% of community-dwelling older Australians involved in a social or support group [31], members of older people-specific social clubs and organizations are expected to be a fair representation of the target population of this project.

Each club/association/group will be approached with a written invitation to participate. Utilizing a snowballing sampling approach, club members will be asked if they could provide a referral to any other club/organization/group that could be contacted with an invitation to participate. Interested clubs/organizations/groups will be asked to circulate the study’s poster among their members in paper or electronic form. Groups that meet in person will be offered an on-site presentation to promote the study and share the invitation to participate.

To facilitate online recruitment, a designated study Facebook page will be created. Administrators of interested Facebook-based groups will be asked to upload the study’s poster on the group’s Facebook page on behalf of the investigator team. Utilizing a snowballing sampling approach will enable study participants to share our study invite among their networks and to refer us to any contacts who they think may have interest in participating.

The first contact with potential participants will be made via one of the three following approaches: (i) during the on-site presentation, (face-to-face contact), (ii) when interested participants contact the investigator responding to the recruitment poster (telephone, email, or via Facebook), (or iii) when a face-to-face contact (prospective participant, club member, or study participant) provides a referral to another prospective participant (telephone or email contact).

Each potential participant will be asked the following three questions to assess if they meet the eligibility criteria: (a) Have you experienced arthritic pain for over 3 months? (b) Are you over 65 years of age? (c) Do you live in the community setting (not in an institutional home like residential aged care facility)? Participants answering “Yes” to all of the three questions will be considered eligible to participate in the study. Those meeting the eligibility criteria will be provided with a brief verbal overview of the study including a clear indication that there is no direct incentive to them in participating in this study. This will be followed by the provision of the written Participant Information and Consent Form. Potential participants will be prompted to ask any questions regarding the implications of participation before signing the consent form. After this time point, potential participants will be advised to get in touch with the study team when they are ready to provide their written consent. This could happen on the same meeting session (or day) of information provision, or could be up to 2 weeks before the end of the recruitment period.

### Sample size

As this is a phase I feasibility study, a sample size calculation is not appropriate. However, consistent with other comparable studies [32, 33], *N* = 30 will be the target recruitment figure.

### Intervention

The intervention to be tested in this study is the free, widely available and downloadable pain self-management app named Rheumatoid Arthritis Information Support and Education (“RAISE”) [34]. The selection was made based on our recent systematic review of the quality and older people-specific usability of all available pain apps identified on the web as of 30 May 2016 [13]. The WebMD Pain Coach app [35] generated the highest quality and usability score [35]; however, this app has recently been removed from the public domain. The app scoring the second highest score RAISE [34] will be selected for evaluation in this study. Written permission has been obtained from the developers of the RAISE app for its use in this study.

### The RAISE app

The RAISE app has been designed by the collaborative effort of the Rheumatology Department of St. James Hospital in Ireland and Arthritis Ireland for the self-management of arthritic pain [36]. The features of the RAISE app could be broadly classed as encompassing the following functions:

- *Assessment and documentation:* The RAISE app offers an option to assess pain in a 0–5 Numeric Rating Scale (NRS) and keeps a time-stamped record of the NRS score. This pain intensity scale can be used as frequently as the user desires. Users can also record their activity level on a 0–5 NRS, and
- *Pain self-management education:* The RAISE app provides education on a range of different topics relating to pain self-management such as a provision of education on pain/pain self-management processes, medication use, and communication with health professionals and pain-related problem-solving. Information on fatigue, sleep, and the management of distress along with CBT pain management instructions on relaxation, goal-setting, and activity pacing (20–30 min session) are provided. Finally, there are videos of stretching, isotonic and aerobic exercise with warmup and cooldown stages with the duration and frequency of exercise also indicated.

### Intervention delivery

A meeting (face-to-face or via telephone) will be organized with all consenting participants in order to download and set up the RAISE app on the participant’s mobile device, provide participants with app training and brief them to use the app for 14 days, and collect the pre-test data (Time 1 (T1)). Participants will be shown all of the features of the app and then guided to navigate through the features of the app until they are comfortable using it. In addition, participants will be advised to contact one of the investigators if they require any assistance in using the app throughout the 14-day period.

A wireless-enabled device (wireless internet dongle) will be purchased and used for downloading the app to the face-to-face meeting participant’s device so that they do not have to use their mobile phone’s data allowance for the app download. If a face-to-face meeting is not feasible, and the participant is willing and able to use their own wireless or mobile internet for the app download, this session will be carried out over the phone (telemeeting). Participants will be advised to use the RAISE app as desired for the next 14 days. It is important to note that this study is not prescriptive in terms of how often participants will use the app. They receive brief training on how to use the intervention app to ensure they are comfortable with using it; however, the frequency with which they use it is up to them.

### Study outcomes

For the purpose of this study, following three preliminary outcomes will be measured before and after the intervention: (1) pain severity and interference, (2) pain self-efficacy—described as confidence people with ongoing pain have in performing activities while in pain [37], and (3) online technology self-efficacy—described as confidence in using various kinds of online technology [38]. In addition to these preliminary outcomes, this study will also measure the following feasibility outcomes: (1) recruitment, refusal, and attrition rates; (2) proportion and patterns of missing data; and (3) ability to recruit 30 participants within 6 months of recruitment commencement.

### Data collection

Data collection will be carried out by one of the investigators who is a registered nurse with a Bachelors of Nursing (Honors) qualification and is currently undertaking a doctoral degree. She has over 5 years experience of working in healthcare research environment across various aged and chronic care trials in the aged care and hospital setting.

Data will be collected using the following questionnaires (refer Table 1):

1. Pain outcomes Case Report Form (CRF)—This CRF includes three valid and reliable outcomes measurement questionnaires, namely the Brief Pain Inventory-short form [39], the short form Pain Self-Efficacy Questionnaire (two-item scale) [40, 41], and the Online Technology Self-Efficacy Questionnaire [38].
  - The Brief Pain Inventory (BPI)-short form—The BPI-sf is a validated, widely used, self-administered questionnaire developed to assess the severity of pain and the impact of pain on daily functions [39].
  - Short form Pain Self-Efficacy Questionnaire (PSEQ-2)—The PSEQ-2 is a valid and reliable two-question tool designed to assess the confidence people with ongoing pain have in performing activities while experiencing unrelieved pain [40, 41]. This tool is deemed to be a robust measure of pain self-efficacy [42].
  - Online Technologies Self-Efficacy Scale (OTSES)—The OTSES is a valid tool that is designed to measure an individual’s self-efficacy in using online technologies [38].
2. Demographic and clinical survey—The survey captures participants’ demographic, social, and health-related information and includes the Charlson Comorbidity Index [43], the Life-Space Assessment questionnaire [44], and the Australian-modified Karnofsky Performance Scale [45].

**Table 1:** Data collection tool and time points

Tool	Day 0 (T1)	Day 15 (T2)
Demographic and clinical survey captures the following:	✓	✘
• Participant’s demographic information, medication use details, social support, technology use pattern, and clinician details		
• Comorbidity: Charlson Comorbidity Index		
• Mobility: Life-Space Assessment questionnaire		
• Performance status: Australian-modified Karnofsky Performance Scale		
Pain outcomes measurement Case Report Form includes the following:	✓	✓
• The Brief Pain Inventory-short form		
• Pain Self-Efficacy Questionnaire-short form (two-item scale)		
• Online Technology Self-Efficacy Questionnaire		

Data collection commenced in July 2018 and will continue for 6 months or until all of the data has been collected for the 30th participants, whichever occurs first.

### Qualitative sub-study

All participants will be invited to take part in a semi-structured telephone interview at the end of the intervention period. The semi-structured interviews will focus on evaluating the acceptability of the intervention. Participants will be specifically asked about the pattern of app use, including frequency and timing, and their experiences, attitudes, and perspectives on integrating the RAISE app into their pain self-management strategy. Participant’s perceptions of the barriers and facilitators to successful use of the intervention app, as well as their willingness and concerns regarding its use, will also be explored. The semi-structured interview will be carried out via telephone. It is anticipated that the interviews will last for approximately 30–45 min. The interviews will be audio recorded and transcribed verbatim for analysis.

### Statistical analysis

This study will utilize the IBM Statistical Package for Social Science (SPSS) software for management and analysis of data. Descriptive statistics will be used to synthesize the sociodemographic data of the participants. Frequencies and percentages will be reported for categorical variables, normally distributed continuous variables will be presented as mean and standard deviations, and median and interquartile range will be reported for non-normally distributed continuous variables. Participant’s pre-test self-reported pain data (pain intensity and pain interference), pain self-efficacy, and online technology self-efficacy will be compared with their post-test reports.

### Qualitative analysis

The qualitative data will be managed using NVivo software. Coding and classification of the data will be carried out to make sense of the collected data and to highlight the features and messages of the semi-structured interviews [46]. Data analysis will be carried out using an inductive process of thematic content analysis. The transcribed interviews will be read and re-read so as to promote immersion in the data and close examination of the interview content. Preliminary themes and sub-themes will be generated and continually refined.

### Data integration and synthesis

Integration of data will take place after the completion of qualitative data collection. The data will be integrated through a triangulation approach [47]. A “coding matrix” will be developed listing the findings from the quantitative and the qualitative study, followed by critical evaluation to find out if the findings from the two studies agree (convergence), if they offer same information on the same issue (complementarity), or if they contradict each other (dissonance) [47]. The aim of this integration process is to develop “meta-themes” that will enable the creation of a composite picture of the whole phenomenon of interest. Based on this integration, a series of recommendations will be made to inform future pain app studies.

### Ethical considerations

The ethical approval for this study was obtained from The University of Notre Dame Australia Ethics Committee (approval number: 017049S). This study is also registered in the Australia New Zealand Clinical Trials Registry (ANZCTR) under the trial ID: ACTRN12617000921381.

### Data management plan

Study data will be recorded in electronic and hard copy form for the purpose of study administration and for the collection of participant data.

#### Electronic recording

An Excel spreadsheet will be created to store participants’ confidential contact information together with their allocated study ID. This will be the only link between each participant and their study ID. This linkage of information is necessary to carry out the post-test data collection, to organize the semi-structured interviews with interested participants, and in cases where a participant later wishes to withdraw from the study and wants their data removed.

The audio recording of the semi-structured interviews will also be transcribed in electronic form (Microsoft Word file). Each interviewee will be provided with a study ID, and any identifiable information that may be present in the audio recording will be de-identified in the transcription process before transferring the data to NVivo software. These files will be stored in the researcher’s password-protected laptop computer and saved within password-protected folders for added security. All data entry work will be carried out by one of the investigators.

#### Hard copy recording

The consent forms of each participant will be obtained in hard copy or paper form. In addition, the pre-post test data will also be collected in hard copy form. The hard copy of the participant’s consents will be stored in a locked cabinet securely and separately from their study data.

All of the data storage will be done in a locked cabinet securely at the principal investigator’s office. The access to the study data will be provided only to the investigator team. At the completion of the study, all study material (electronic and hard copy) will be reconciled and stored as per the relevant state regulation regarding research data retention and disposal of that time.

### Dissemination

Data analysis will start immediately after the data collection period. The result of this study will be published in peer-reviewed journals and presented in relevant conferences.

## Discussion

This phase I feasibility study will make an important contribution to determining the feasibility and acceptability and building the evidence base concerning the use of pain self-management apps for older people living with arthritic pain. In addition to being the first study to evaluate the feasibility and acceptability of a pain self-management app in the older population, this study will also provide preliminary insights into the impact of a self-management app in older people’s pain and self-efficacy-related outcomes. Given the globally aging population, the prevalence of chronic pain, an upward trend of smartphone adoption among older people, and the paucity of evidence in the area of pain self-management apps among older people, this study aims to address an important evidence gap.

Several limitations of this study should be considered. Firstly, this study is not prescriptive in terms of how often participants are to use the app. They will receive a brief training on how to use the intervention app to ensure they are comfortable with using it; however, the frequency with which they use it is up to them. This could lead to non-uniform app use pattern among the participants. However, as we intend to evaluate the feasibility and acceptability of the pain self-management app as it resembles the “real-world” situation, the varying pattern of app use will be considered and evaluated in a qualitative sub-study with the participants (reported elsewhere). Secondly, there is a possibility of selection bias as we aim to recruit via social clubs and associations which could exclude those who are less socially active and involved. To minimize this potential, we have also opted for snowballing recruitment approach where referrals to non-members from participating clubs/individuals are followed-up adequately for inclusion. However, with the utilization of snowballing sampling approach, it will be challenging to appropriately capture the refusal rate resulting from snowballing, and this will be a limitation of this study.

Despite the above-noted limitations, findings from this study will be relevant in informing future arthritic pain self-management research and similar app development endeavors. The findings of this study are expected to help develop a set of recommendations that could inform policymakers, clinicians, app developers, and consumers when developing, selecting, or implementing a pain self-management app.

## References

[1] World Health Organization. Global Health and Aging. National Institutes of Health publication no. 11–7737. 2011. Retrieved from http://www.who.int/ageing/publications/global_health.pdf.

[2] Patel KV (2013). Prevalence and impact of pain among older adults in the United States: findings from the 2011 National Health and Aging Trends Study. PAIN®.

[3] Australian Bureau of Statistics. Arthritis and osteoporosis, in Australian Health Survey: first results, 2011–2012. Canberra: Australian Bureau of Statistics; 2012.

[4] March LM, Bachmeier CJM (1997). 10 economics of osteoarthritis: a global perspective. Baillières Clin Rheumatol.

[5] Nicholas MK (2012). Is adherence to pain self-management strategies associated with improved pain, depression and disability in those with disabling chronic pain?. Eur J Pain.

[6] Schofield P (2014). Systematically searching for and assessing the literature for self-management of chronic pain: a lay users’ perspective. BMC Geriatr.

[7] Lorig K, Holman HR (2003). Self-management education: history, definition, outcomes, and mechanisms. Ann Behav Med.

[8] Pew Research Center (2017). Health fact sheet.

[9] Pew Research Center. Older adults and technology use. 2014. Available: http://www.pewinternet.org/2014/04/03/older-adults-and-technology-use/.

[10] Pew Research Center. Tech adoption climbs among older adults. 2017. Available: https://www.pewinternet.org/2017/05/17/tech-adoption-climbs-among-older-adults/. Accessed 17 July 2017.

[11] Boulos MNK (2014). Mobile medical and health apps: state of the art, concerns, regulatory control and certification. Online J Public Health Inform.

[12] Ventola CL (2014). Mobile devices and apps for health care professionals: uses and benefits. Pharm Ther.

[13] Bhattarai P, Newton-John TRO, Phillips JL (2017). Quality and usability of arthritic pain self-management apps for older adults: a systematic review. Pain Med.

[14] Lalloo C (2015). “There’s a pain app for that”: review of patient-targeted smartphone applications for pain management. Clin J Pain.

[15] Wallace LS, Dhingra LK (2013). A systematic review of smartphone applications for chronic pain available for download in the United States. J Opioid Manag.

[16] Reynoldson C (2014). Assessing the quality and usability of smartphone apps for pain self-management. Pain Med.

[17] Rosser BA, Eccleston C (2011). Smartphone applications for pain management. J Telemed Telecare.

[18] Stinson JN (2013). Development and testing of a multidimensional iPhone pain assessment application for adolescents with cancer. J Med Internet Res.

[19] Kristjánsdóttir ÓB (2013). A smartphone-based intervention with diaries and therapist-feedback to reduce catastrophizing and increase functioning in women with chronic widespread pain: randomized controlled trial. J Med Internet Res.

[20] Wesley KM, Fizur PJ (2015). A review of mobile applications to help adolescent and young adult cancer patients. Adolesc Health Med Ther.

[21] Van Het Reve E (2014). Tablet-based strength-balance training to motivate and improve adherence to exercise in independently living older people: part 2 of a phase II preclinical exploratory trial. J Med Internet Res.

[22] Yamada M, et al. Using a smartphone while walking: a measure of dual-tasking ability as a falls risk assessment tool. Age Ageing. 2011;40(4):516–9. 10.1093/ageing/afr039 21593058

[23] Currie M, Philip LJ, Roberts A (2015). Attitudes towards the use and acceptance of eHealth technologies: a case study of older adults living with chronic pain and implications for rural healthcare. BMC Health Serv Res.

[24] Parker SJ (2013). Older adults are mobile too!Identifying the barriers and facilitators to older adults’ use of mHealth for pain management. BMC Geriatr.

[25] Campbell M (2000). Framework for design and evaluation of complex interventions to improve health. BMJ.

[26] Craig P (2008). Developing and evaluating complex interventions: the new Medical Research Council guidance. BMJ.

[27] Bowen DJ (2009). How we design feasibility studies. Am J Prev Med.

[28] Eldridge SM (2016). Defining feasibility and pilot studies in preparation for randomised controlled trials: development of a conceptual framework. PLoS One.

[29] Tickle-Degnen L (2013). Nuts and bolts of conducting feasibility studies. Am J Occup Ther.

[30] Patford J, Breen H (2009). Homes away from home: registered clubs as leisure providers for older people living in the Tweed Heads region of Australia. Ann Leis Res.

[31] Welfare, A.I.o.H.a. and Welfare (2007). Older Australia at a glance.

[32] Paul L (2017). Increasing physical activity in older adults using STARFISH, an interactive smartphone application (app); a pilot study. J Rehabil Assistive Technol Eng.

[33] Silveira P (2013). Tablet-based strength-balance training to motivate and improve adherence to exercise in independently living older people: a phase II preclinical exploratory trial. J Med Internet Res.

[34] St James’s Hospital and Arthritis Ireland. RAISE V1.0.3. [Mobile application software] 2015; Available from: http://www.apple.com/au/itunes/.

[35] WebMD (2016). WebMD Pain Coach V1.3 [Mobile application software].

[36] WebMD (2015). WebMD Pain Coach App.

[37] Nicholas MK (2007). The pain self-efficacy questionnaire: taking pain into account. Eur J Pain.

[38] Miltiadou M, Yu CH (2000). Validation of the Online Technologies Self-Efficacy Scale (OTSES).

[39] Mendoza T (2006). Reliability and validity of a modified Brief Pain Inventory short form in patients with osteoarthritis. Eur J Pain.

[40] Tonkin L (2008). The pain self-efficacy questionnaire. Aust J Physiother.

[41] Miles CL (2011). Measuring pain self-efficacy. Clin J Pain.

[42] Nicholas MK, McGuire BE, Asghari A (2015). A 2-item short form of the Pain Self-Efficacy Questionnaire: development and psychometric evaluation of PSEQ-2. J Pain.

[43] Charlson ME (1987). A new method of classifying prognostic comorbidity in longitudinal studies: development and validation. J Chronic Dis.

[44] Stalvey BT (1999). The Life Space Questionnaire: a measure of the extent of mobility of older adults. J Appl Gerontol.

[45] Abernethy AP (2005). The Australia-modified Karnofsky Performance Status (AKPS) scale: a revised scale for contemporary palliative care clinical practice [ISRCTN81117481]. BMC Palliat Care.

[46] Miles MB, Huberman AM, Saldana J. Qualitative data analysis: a methods sourcebook. SAGE Publications; 2013.

[47] O’Cathain A, Murphy E, Nicholl J (2010). Three techniques for integrating data in mixed methods studies. BMJ.

